# A Fishy Novel, by a Texas Doctor

**Published:** 1895-11

**Authors:** 


### EDITORIAL DEPARTMENT.

F. E. DANIEL, M. D., Editor.
S. E. HUDSON, M. D., Managing Editor.
A. J. SMITH. M. D., Galveston, Associate Editor.

EDITORIAL STAFF:

PROF. J. E. THOMPSON, M. D., Texas Medical College, Galveston; Surgery.
PROF. WM. KEILLER, M. D., Texas Medical College, Galveston; Obstetrics and Gynecology.
PROF. DAVID CERNA, M. D., Texas Medical College, Galveston; Therapeutics.
PROF. A. J. SMITH, M. D., Texas Medical College, Galveston; Medicine.
DR. R. H. L. BIBB, Saltillo, Mexico; Foreign Correspondent.

Official organ of the West Texas Medical Association, the Houston District Medical Association, the Austin District Medical Society, the Galveston County Medical Society, and several others.

### A FISHY NOVEL, BY A TEXAS DOCTOR.

Dr. J. W. Carhart, who lately removed from Lampasas to La Grange, and who is well known to the medical profession of the United States as one of the organizers of the *Pan-American Medical Congress*, and one of its officers, has written a small book, a novel. In fact, he has written several small books,—but the one in question is the only one we ever read, and we read this one out of curiosity, as doubtless others will do,—because of its remarkable character; and because, too, the author is one of the Texas doctors.

It is called "Norma Trist; A Story of Inversion of the Sexes." ("Abnormal Twist" would have been a more appropriate name.) It is based upon the Alice Mitchell episode, of which it is an almost exact counterpart. The scene is laid in La Grange. It is the story of a finely-developed young girl, a pink of perfection, morally and intellectually; the daughter of a rich widow. During her father's lifetime, she being the only child, was made his companion in all things, and as she rode horseback and, sometimes, "straddle,'—when she was a child,—she developed masculine traits to some extent, and this training, the author makes responsible, later on, for the perversion or inversion of the sexual instinct. She is passive and indifferent to all male society, and has a horror of the idea of loving a man. There is a young fellow, by name "Frank Artman," who is desperately in love with her, and has been, since childhood. He wooes her in vain.

She conceives a violent passion of some kind,—we can not say "love,"—for her music-teacher, a buxom young widow, with a high-sounding French name,—"Mrs. Marie LaMoreaux"; but nothing is said of it, and no one knows of it till Norma is sent off to boarding-school at Maplewood Institute in Massachusetts. From Maplewood she writes letters to Mrs. LaMoreaux of a very remarkable character. One of these letters accidentally falls into the hands of the Faculty, and creates great consternation and dismay; they do not know what to make of it. The author gives these letters in full. It is a rapt, ecstatic description of the lascivious emotions excited in the young girl's breast at thoughts of her distant "beloved." Later, the beloved makes Norma a present of a fur cape. This fur cape, Norma declares in her next letter, is her *fetich*,* and says that the touch of it excites in her the strongest and most delightful sensations. These erotic sensations culminate, we are led to infer, in an orgasm, and the author of the book faithfully describes, or makes her describe them; a delectable morsel for the unsophisticated of the general public. The girl graduates with first honors, and goes home. She finds her beloved engaged to be married to a Spanish officer, and, furious with jealousy, she attempts to assassinate her on the street. She is sent to an insane asylum at Austin, and after a sojourn of some months, is discharged, the superintendent pronouncing her not insane. She is then tried for assault and intent to kill. The jury can not agree, and the case seems to be indefinitely postponed.

At the trial "a physician" is introduced "as an expert." This physician the author calls "Dr. Jasper," and says he is a very distinguished gentleman, who has recently located at La Grange, and of whom all the other physicians are envious, on account of his great ability and his distinguished reputation; he having been "one of the organizers of the *Pan-*

---

[* Fetich: any material object regarded with veneration or awe. FETICH, FETICHISM.

The term applied by Binet to the sexual perversion exhibited by collectors of napkins, shoes, etc. He maintains that these articles play here the part of the fetich in early theology. The favors given by the women to the knights in the middle ages, were both tokens of remembrance, and sexual excitants of satisfaction. Fetichism is the association of lust with the idea of certain portions of the female person, or with certain articles of female attire. It is designated as dress-f; hair-f; glove-f; shoe-f, etc.]—Gould's Dictionary of Medicine, 465.

*American Medical Congress*, and one of its officers; a member of the Texas State Medical Association, who has contributed many valuable papers to its volume of Transactions; a man of uncertain age,—of distinguished presence,—a calm and thoughtful manner, such as usually distinguishes men of science,''—or words to that effect;—in fact, a paragon and model in all the proprieties, as well as in all scientific matters. This great doctor proceeds to enlighten judge and jury on *psychopathia sexualis*, quotes a book by that title by Craft-Ebing, and also quotes Notzing: It is nothing new to him; he can *cure it*, right out of hand! This trial is faithfully reported, and reminds one of a trial in a police court reported in the *Police Gazette*. Nothing is omitted. "A last question" says the prosecuting attorney, "he wants to ask Norma": "Are your relations with Madame LaMoreaux satisfying?" (Page 212.) In reply Norma says: "I understand you to mean satisfaction of the erotic desire?" "That is what I I mean," says the attorney. "I have no hesitatancy in answering frankly and freely that my love for and relations with Marie afforded the highest and profoundest satisfaction of which the entire human being is capable in the realm of human love," replied this modest young lady—"pure carbon"—the author calls her.

(As nothing has been said in the book of any relations other than those of close social intercourse--the reader is left very considerately to infer what those relations might be; the only departure in the book from "realism" gone mad.*)

---

* For fear I should do the author injustice, unwittingly, in drawing the inference mentioned above, that the fetich excited sexual desire to the point of orgasm, and that "satisfaction" meant this, I wrote to the author, asking what was meant by "relations," and if readers were to infer that there had been bodily contact, embraces, caresses; and if the pleasure the girl described was sexual desire gratified. I received a letter, from which the following extracts are made. They are given here to show that I did not misconstrue his meaning. The doctor says, in reply to my inquiry: "You will find in Norma's letter to Mrs. LaMoreaux, on page 52-3, the following language: "I am happy only when I think of you and fancy I am in your arms, hugging you to my bosom. You are the idol of my life; the inspiration of my thoughts—the only stimulus of my amorous feelings. How happy the hours in the past, when, unsuspected, I could yield myself in your embraces to the full sway of passion's convulsive joys."

Again, the doctor reminds me that when Norma had stabbed the woman, she exclaims, "Oh, how I loved her. I have killed her. This blood [smelling her hands], as I smell of it, makes me feel so strange; makes me feel as when I had her in my arms and hugged her to my bosom." "The above

Finally, this great Dr. Jasper, whom it will not be very difficult to recognize in the person of the author, drawn and colored from his own standpoint of observation, and endowed with all the excellencies, gets consent of the mother to treat Norma by hypnotic suggestion, and does treat her. He "suggests" that she forget Mrs. LaMoreaux and love Frank, which she proceeds to do; and all is lovely,—even to the cottage with the morning-glory vines on the gallery, and the two sunny-headed children;—an anachronism, by the bye, as the great Dr. Jasper has only resided in La Grange about a year.

We confess we read Dr. Carhart's book with feelings both of surprise and indignation. Surprise, that so good a man and physician as we have heretofore believed Dr. Carhart to be, should commit such a rash breach of propriety, to say the least,—as to put such a story upon the market for the general reader. Indignant, that one of our profession,—a man who, by every consideration of his age, his antecedents, and his record since his connection with the Texas profession should sustain every effort in behalf of pure morals, and co-operate with medical journalists and teachers in the endeavor to suppress or eliminate the indecent in literature, should have made himself such a conspicuous exception, and brought obloquy upon his associates. We can conceive of no motive—other than the hope of pecuniary gain,—a most unworthy one—which could have actuated him in committing such an outrage upon decency; unless, perhaps, he thought to advertise himself as a physician skilled in the new fad—hypnotism;—still more unworthy. Why, the book is scarcely fit for a doctor to read. Quoting a critic in the *Literary Digest*, who roasts Thomas Hardy for a publication of a similar

---

quotations," continues the letter, "embrace all that I can now recall that might be construed as having special allusion to anything like physical contact or 'climax' or 'orgasm,' in the relations of the two women. Her contact with her fetich implies sexual excitement, as she says in her letter, page 66." This erotic delight in furs is something entirely different from æsthetic pleasure." On page 67 she says: "How this peculiar impression on the tactile nerves is related to sexual instinct, is a perfect enigma to me." * * "Fur, per se, arouses sensuality in me; how, I can not explain." The doctor then adds: "Orgasm is frequently had by the smell, and even the taste of the blood of the object sexually loved; FOR THIS REASON Norma smells the blood on her hands, and if you wish to push it that far, I would admit that she had orgasm, but the language does not imply that." [Well, I should think it does, to a man up a tree.—ED.]

nature (and Hardy is not a doctor, either), we hold that "nudity for nudity's sake, and delineation of erotic desire as an appeal to sensuousness, are simply nastiness, and debase the artist and the author into panders who debauch public sentiment for the sake of gain." Should the book be read, it will do incalculable harm. No possible good can come of it. We see nothing in it to commend, but everything to condemn, and we can not do so in language too strong. The sexual perversion part of it is all there is in it, and its excuse for being is that it is a scientific subject treated by a man who pretends to science, in a popular work of fiction. But even that is poorly portrayed. If a reader desires any literature of the kind, let him get the report of the Alice Mitchell trial, with the expert opinion of Professors Callender, Sim and others; it is infinitely more edifying and interesting. All the rest is mere stuffing, or stuff, and a poor quality at that; for instance, the author makes Frank find a hidden treasure,—for which there was no occasion.

Dr. Carhart who, by the bye, we learn, has associated with him in the venture and the profits, a Baptist preacher, has done himself no credit in publishing such a book, and his friends will blush for him in very shame and humiliation. He should have put his talents and his time to nobler work. We understand he will shortly publish another of a similar character, but worse, if possible. For very shame!

The book can well be classed as obscene, and should be dealt with by the authorities as such; its sale prohibited, and transmission through the mails denied it.

[We feel like apologizing to our readers for devoting so much space to so unworthy, though important a subject, but we felt we could hardly do either the subject or the author justice in less.]

